# Stepwise Targeted Matching Strategy for Comprehensive Profiling of Xanthohumol Metabolites In Vivo and In Vitro Using UHPLC-Q-Exactive Orbitrap Mass Spectrometer

**DOI:** 10.3390/molecules28135168

**Published:** 2023-07-02

**Authors:** Xiaoqing Yuan, Hong Wang, Shuyi Song, Lili Qiu, Xianming Lan, Pingping Dong, Jiayu Zhang

**Affiliations:** 1 College of Pharmacy, Binzhou Medical University, Yantai 264003, China; 15064369212@163.com (X.Y.);; 2 College of Life Sciences, Shandong Agricultural University, Taian 271018, China; wanghong20201002@163.com; 3 Department of Medicine, Binzhou Polytechnic College, Binzhou 256600, China; 4 State Key Laboratory for Quality Research of Chinese Medicines, College of Pharmacy, Macau University of Science and Technology, Macao 999078, China

**Keywords:** xanthohumol, UHPLC-Q-Exactive Mass Spectrometer, SMM, stepwise targeted matching strategy

## Abstract

Xanthohumol (XN), a natural prenylated flavonoid extracted and isolated from the hop plant (*Humulus lupulus*), possesses diverse pharmacological activities. Although the metabolites of XN have been investigated in the previous study, a comprehensive metabolic profile has been insufficient in vivo or in vitro until now. The current study was aimed at systematically elucidating the metabolic pathways of XN after oral administration to rats. Herein, a UHPLC-Q-Exactive Orbitrap MS was adopted for the potential metabolites detection. A stepwise targeted matching strategy for the overall identification of XN metabolites was proposed. A metabolic net (53 metabolites included) on XN in vivo and in vitro, as well as the metabolic profile investigation, were designed, preferably characterizing XN metabolites in rat plasma, urine, liver, liver microsomes, and feces. On the basis of a stepwise targeted matching strategy, the net showed that major in vivo metabolic pathways of XN in rats include glucuronidation, sulfation, methylation, demethylation, hydrogenation, dehydrogenation, hydroxylation, and so on. The proposed metabolic pathways in this research will provide essential data for further pharmaceutical studies of prenylated flavonoids and lay the foundation for further toxicity and safety studies.

## 1. Introduction

**Figure 1:**
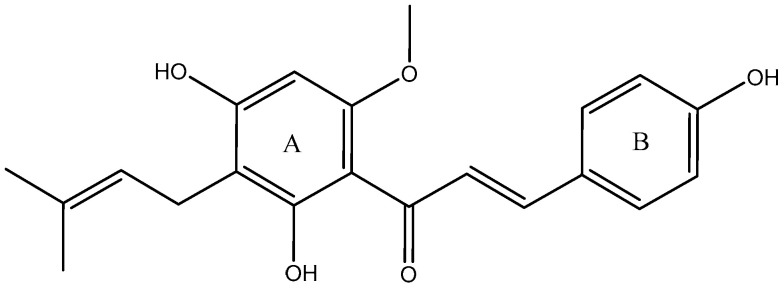
Chemical structure of XN.

Xanthohumol (XN, Figure 1), the most abundant prenylated flavonoid with 0.1–1.0% of dry weight in hop plants (*Humulus lupulus*) [1], can be isolated from the female inflorescences (cones), acting as a preservative to give beer its unique aroma and flavor [2,3]. XN is also a constituent of beer, a major dietary source of prenylated flavonoids, where it has been found at concentrations up to 0.96 mg/L [4]. It is reported that XN has been widely used in the treatment of cardiovascular diseases [5], metabolic syndrome [6], Alzheimer’s disease (AD) [7], diabetes and diabetic complications [8], and bone resorption [9]. Additionally, XN has been patented as a drug for osteoporosis treatment [10]. According to previous reports, its pharmacological actions were mainly attributed to its abilities to scavenge oxygen free radicals [5], inhibit tumor angiogenesis [11], reduce lipid peroxidation [12], and so on.

Research on XN’s metabolites in vivo and in vitro has been carried out. For example, Yilmazer M. et al. have discovered that XN can be biotransformed to glucuronides, hydroxylated metabolites, and cyclic dehydro-metabolites in rats and human liver microsomes [13,14]. Investigations using human liver microsomes showed that hydroxylation of a prenyl methyl group is the primary route of the oxidative metabolism, forming hydroxylated metabolites of XN [15]. XN can also be converted to desmethylxanthohumol (DMX) directly [16]. When XN was fed to rats at a dose of 1000 mg/kg body weight, feces was the major route of excretion [17,18], where 22 XN metabolites were identified [19]. Although the metabolism of XN has been reported, it has only been detected in a few metabolites. There were shortages in comprehensive descriptions of XN’s metabolites and their related descriptions in the above analysis.

Metabolic studies in vivo and in vitro have played an increasingly important role in clarifying the mechanism of action of the drugs and providing a crucial basis to guide clinical medication recommendations [20,21,22,23]. In the last few decades, with the development of various data acquisition methods, ultra-high-performance liquid chromatography coupled with mass spectrometry (UHPLC-MS), especially high-resolution mass spectrometry (HRMS), has exhibited extraordinary performance for metabolite detection due to its high speed and detection sensitivity [24,25,26]. However, numerous metabolite signals from the full-scan mass chromatograms obtained from liquid chromatography mass spectrometry (LC-MS) analysis are likely to be submerged by interferences from the background or matrix without comprehensive metabolite scanning. Additionally, there is no denying that multiple data-mining techniques, merely based on neutral loss filter (NLF), high-resolution extracted ion chromatography (HREIC), multiple mass defect filtering (MMDF), and the use of diagnostic product ions (DPIs), could not fully cover comprehensive metabolite profiling. In this respect, designed to clarify possible XN biotransformation pathways in vivo and in vitro, a stepwise targeted matching strategy based on the full-scan data acquisition method coupled with multiple data-mining techniques, followed by the discovery of SMMC centered on SMM, was proposed. The strategy could be employed as a rapid and effective technique to screen and identify XN targeted constituents.

## 2. Results

### 2.1. The Establishment of a Stepwise Targeted Matching Strategy Based on SMM and SMMC

**Figure 2:**
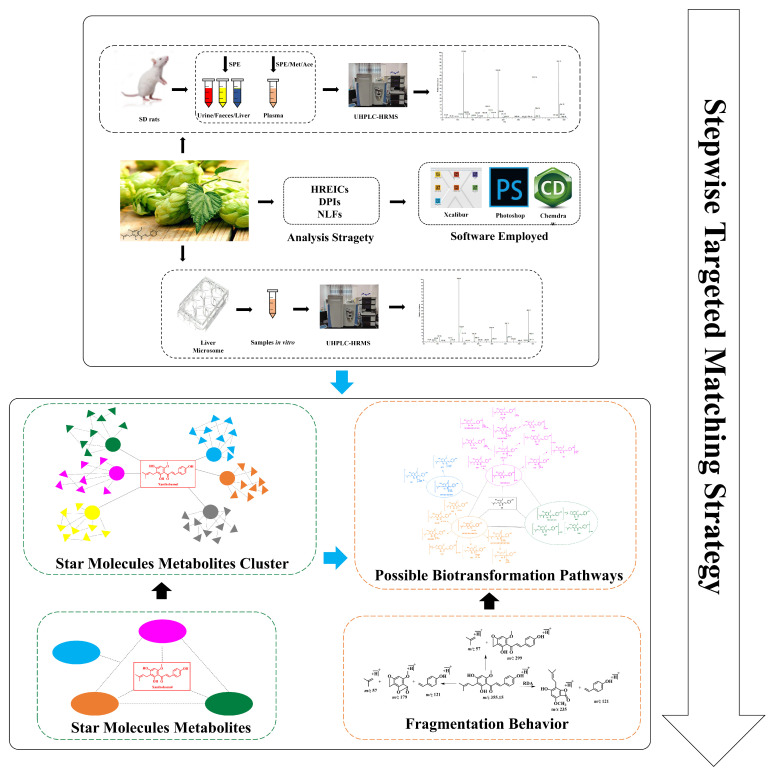
Summary diagram of the developed strategy and methodology.

A systematic and efficient strategy (Figure 2) was established for the comprehensive screening and characterization of XN metabolites in SD rats’ urine, plasma, feces, liver tissues, and liver microsomes using UHPLC-Q-Exactive Orbitrap MS, and then we dealt with the collection of data by post-acquisition data-mining processing techniques.

The liver has long been recognized as the primary site of drug metabolism in the body. Drug metabolism is classified into phase I and phase II reactions. Phase I metabolism usually does not result in a large change in the molecular weight or water solubility of the substrate but is of great importance because oxidative reactions add or expose sites where phase II metabolism can subsequently occur. In contrast, phase II conjugation typically results in an appreciable increase in molecular weight and water solubility [6]. By reviewing the literature and comparing the data of our analysis, we found that XN and its metabolites are found in the free form or as conjugates (mainly as glucuronidation and sulfonation) [13,14,27] in phase II. Therefore, we focused on obtaining the glucuronidation and sulfation metabolites, which we called “Star Molecules Metabolites” (SMM), and a XN “Star Molecules Metabolites Cluster” (SMMC and SMMC1: XN sulfation metabolites, SMMC2: XN glucuronidation metabolites, and SMMC3: XN sulfation together with glucuronidation metabolites) was subsequently depicted on the basis of SMM.

**Figure 3:**
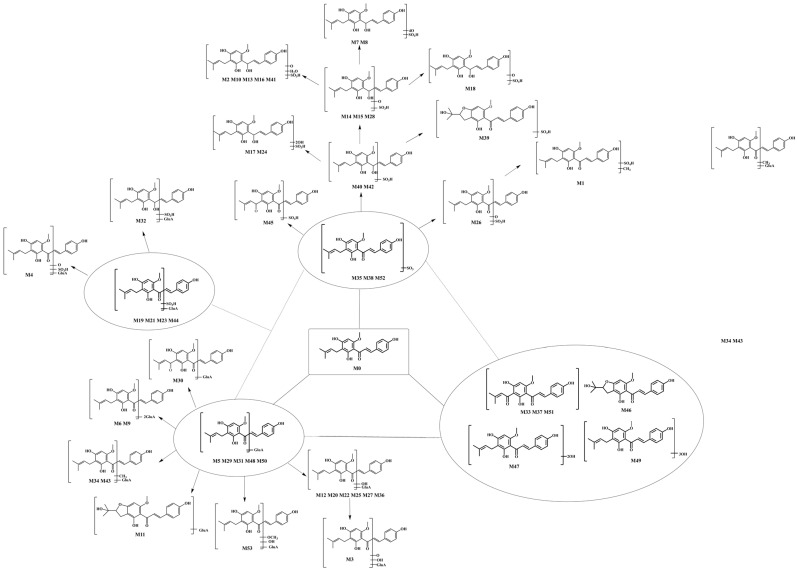
The proposed biotransformation pathways of XN in vivo and in vitro.

Additionally, in this study, NLFs and DPIs were applied to data screening, which could intelligently filter the actual background noise and matrix-related ions from drug-related ions according to the accurate mass measurement of their [M − H]^−^ ions. Then, we inferred the possible metabolic pathways on the basis of the combination of the structural characteristics, literature, and various databases of XN. Thermo Xcalibur 2.1 software was used to acquire their retention times, accurately determine molecular weight, and secondary fragment ion information by screening the candidate compounds, which could be applied to the subsequent structural elucidation and metabolite classification. Eventually, 53 metabolites were identified on the basis of “SMMC”, and the intermediates, together with the final metabolites, formed a metabolic network that centered on XN, accompanying glucuronidation, sulfation, and the other metabolites. The possible metabolic processes of XN in vitro and in vivo were described via a stepwise targeted matching strategy (Figure 3).

### 2.2. Fragmentation Pattern Analysis and DPI Determination

**Figure 4:**
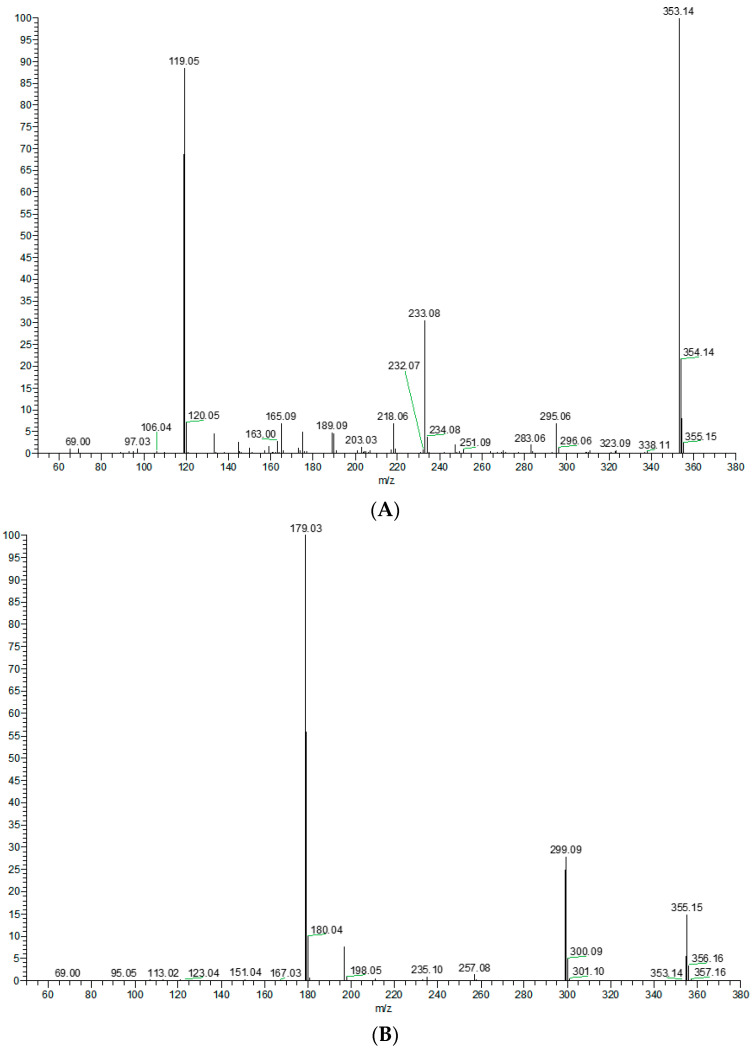

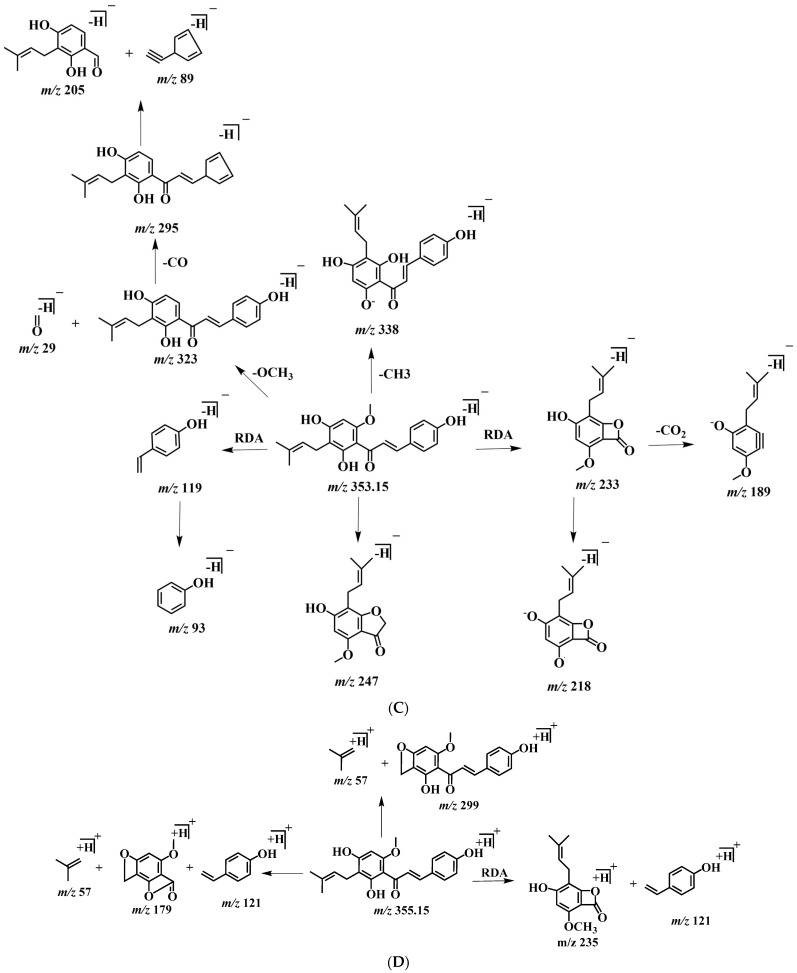
ESI-MS/MS spectra information and cleavage pathways of XN. (**A**) The ESI-MS/MS spectra of XN in positive ion mode. (**B**) The ESI-MS/MS spectra of XN in negative ion mode. (**C**) The mass fragmentation behavior of XN in negative ion mode. (**D**) The mass fragmentation behavior of XN in positive ion mode.

In order to better characterize the metabolites of rats in vivo and in vitro and provide theoretical support for the following rapid analysis, DPIs, such as CO, CH_3_, and CH_3_OH, and NLFs, such as *m*/*z* 233, *m*/*z* 119, and *m*/*z* 249 of XN, were summarized on the basis of the mass spectrometric cracking rules reported in the literature and the cracking information of reference substances. XN yielded its [M + H]^+^ and [M − H]^−^ ions at *m*/*z* 355.15323 and *m*/*z* 353.13861, respectively, in the ESI-MS^2^ spectra. The ESI-MS/MS spectra of XN are shown in Figure 4: (1) Important fragment ions were formed by the retro Diels–Alder (RDA) reaction, yielding a pair of complementary product ions at *m*/*z* 233 (the A ring) and *m*/*z* 119 (the B ring) [17]. Fragment ions such as those at *m*/*z* 189, shown by the loss of CO_2_, and *m*/*z* 93 showed that RDA reaction cracking further occurred. A fragment ion at *m*/*z* 218 was generated due to the loss of the CH_3_ at *m*/*z* 233; and (2) the formation of fragment ions at *m*/*z* 323 and *m*/*z* 338 was 30 Da and 15 Da smaller than XN, respectively, indicating the reaction of demethoxylation and demethylation took place. Based on the preliminary judgment of the addition and subtraction of characteristic fragments such as *m*/*z* 353 ± nx, *m*/*z* 233 ± nx, *m*/*z* 299 ± nx, *m*/*z* 179 ± nx, and *m*/*z* 249 ± nx (x = molecular weight of substituent groups; n = the number of substituent groups), a rough acquisition of the XN metabolites was initially formulated.

### 2.3. Detection and Structural Elucidation of XN Metabolites

There are a total of 53 metabolites detected and identified in the urine, plasma, feces, liver, and liver microsomes of SD rats using a UHPLC-Q-Exactive Orbitrap mass spectrometer. The chromatographic and MS data of these detected metabolites are summarized in Table 1.

#### 2.3.1. Identification of SMMC1

**Figure 5:**
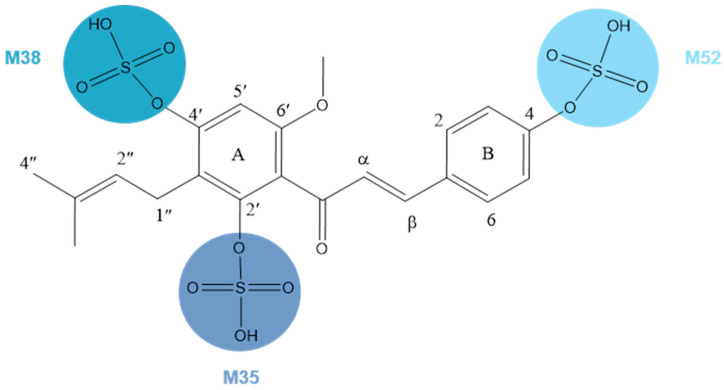
Chemical structure of **M35**, **M38**, and **M52**.

**M35**, **M38**, and **M52**, 80 Da larger than XN, possessed the same theoretical [M + H]^+^/[M − H]^−^ ions at *m*/*z* 435.11191 (C_21_H_23_O_8_S, error ≤ ±5 ppm) and *m*/*z* 433.09626 (C_21_H_21_O_8_S, error ≤ ±5 ppm) in both positive and negative ion modes correspondingly. In the ESI-MS/MS spectrum, a neutral loss of 80 Da (*m*/*z* 433 → *m*/*z* 353) was observed, which indicated the occurrence of a sulfonation reaction. Additionally, the negative ion fragment at *m*/*z* 119 as well as the DPIs for sulfation at *m*/*z* 80 confirmed our deduction. The same is true for the positive ion fragments, DPIs such as *m*/*z* 435, *m*/*z* 355, *m*/*z* 121, and *m*/*z* 235. Furthermore, the substituted sites of SO_3_ were determined by comparing their Clog*P* values and retention times. Compounds with a higher Clog*P* value generally exhibit longer retention times in reversed-phase separation mode [28], thus **M35**, **M38**, and **M52** were finally deduced as XN-2′-SO_3_H, XN-4′-SO_3_H, and XN-4-SO_3_H, respectively, (Figure 5).

**M26** possessed its deprotonated molecular ion at *m*/*z* 449.09008 (C_21_H_21_O_9_S, mass error 0.071 ppm) with a retention time of 6.77 min. It was 16 Da higher than **M35** in negative ion mode. It was preliminarily assigned as hydroxyxanthohumol according to the obtained HRMS data. The DPIs at *m*/*z* 369 [M-H-SO_3_]^−^ and at *m*/*z* 353 [M-H-SO_3_-O]^−^ were attributed to the above analysis. In its ESI-MS/MS spectrum, the fragment ions at *m*/*z* 96 (449 → 353) and *m*/*z* 80 (449 → 369) further provided evidence for hydroxyl and sulfate group identification. Thus, **M26** was identified as the product of the combination of hydroxylation and sulfation.

Isomers **M17** and **M24**, which were, respectively, eluted at 6.36 and 6.64 min, exhibited the same theoretical [M − H]^−^ ions at 465.08499 (C_21_H_21_O_10_S, error ≤ ±5 ppm). They were both 32 Da (2 × 16 Da) larger than **M0**, indicating an oxidation reaction occurred. Thus, **M17** and **M24** were identified as the sulfation products, with 2H being hydroxylated. **M7** and **M8**, 64 Da larger than **M35**, with the theoretical [M − H]^−^ at *m*/*z* 497.07482 (C_21_H_21_O_12_S, mass error < 5 ppm), were eluted at 5.11 min and 5.50 min, respectively. The fragment ion at *m*/*z* 417 [M-H-SO_3_]^−^ implied the appearance of sulfation; similarly, we tentatively deduced **M7** and **M8** were oxidation and sulfation XN.

**M2**, **M10**, **M13**, **M16**, and **M41** generated the [M − H]^−^ ion at *m*/*z* 467.10064 (C_21_H_23_O_10_S, mass error < 2 ppm) with retention times of 5.61 min, 5.96 min, 6.32 min, and 8.87 min, respectively. They were 2 Da larger than **M17** and **M24** in negative ion mode, indicating they could be hydrogenation products of XN-O-sulfation. The appearance of an ion at *m*/*z* 119 implied that the reaction above was conducted on the A-ring rather than the B-ring. Moreover, the DPIs at *m*/*z* 249 supported our discussion.

**M14**, **M15**, and **M28** yielded their significant [M − H]^−^ ions at *m*/*z* 451.10573 (C_21_H_23_O_9_S, mass errors of 0.157, 0.090, and 0.511 ppm), which were eluted at 6.15 min, 6.28 min, and 6.90 min, respectively. The hydrogenation reaction could be deduced to occur because they were 2 Da larger that of **M26**. In their ESI-MS/MS spectra, DPIs at *m*/*z* 371, *m*/*z* 369, and *m*/*z* 119 were generated by the subsequent loss of [M-H-SO_3_]^−^, [M-H-SO_3_-2H]^−^, and C_8_H_8_O. Thus, both **M15** and **M28** were tentatively characterized as hydrogenation products of **M26**.

**M18** exposed 4 Da larger metabolites than the metabolites of the XN, combining hydroxylation and sulfation. Thus, the hydrogenation product was deduced, with an addition reaction occurring on two of the three double bonds. The DPIs at *m*/*z* 455 and at *m*/*z* 375 provided evidence for our diagnosis. What is more, the ions at *m*/*z* 97 [SO_3_H+H+O]^+^ and at *m*/*z* 80 [SO_3_H]^+^ suggested the presence of a sulfuric acid ester group, which indicated that **M18** was a metabolite combining hydroxylation, sulfation, and hydrogenation.

**M40** and **M42**, 2 Da larger than sulfated XN, afforded the deprotonated molecular ions at *m*/*z* 435.11081, revealing that an in vivo hydrogenation reaction might have occurred. Fragments of negative ions at *m*/*z* 355 [M-H-SO_3_]^−^ and *m*/*z* 353 [M-H-SO_3_-2H]^−^ support our deduction. Thus, **M40** and **M42** were identified as the sulfated and hydrogenated products of XN.

#### 2.3.2. Identification of SMMC2

**M6** and **M9**, showed the same theoretical [M + H]^+^/[M − H]^−^ at *m*/*z* 707.21818 (C_33_H_39_O_17_, error < 2 ppm) and *m*/*z* 705.20253 (C_33_H_37_O_17_, error < 2 ppm). **M6** and **M9** were eluted at 5.11 min and 5.59 min, respectively, and possessed identical DPIs at *m*/*z* 529 and *m*/*z* 353, which were considered to be integrated with another glucuronide following the glucuronidation products (705 → 529 → 353). Moreover, other fragment ions, such as *m*/*z* 119 and *m*/*z* 233, confirmed our deduction. The same was also appearing in corresponding [M + H]^+^ ions, such as DPIs at *m*/*z* 707, *m*/*z* 531, *m*/*z* 355, *m*/*z* 179, *m*/*z* 235, and so on. As we can see from the Supplementary Materials, **M9** abundantly appeared in plasma samples, whether the plasma samples were pretreated with SPE, methanol, or acetonitrile. The appearance of XN in plasma provided a basis for the follow-up study on the distribution of drugs in vivo and favorable theoretical support for the study of its pharmacological effects.

**M5**, **M29**, **M31**, **M48**, and **M50** showed the same theoretical [M + H]^+^/[M − H]^−^ at *m*/*z* 531.18609 (C_27_H_31_O_11_, error ≤ 2 ppm) or *m*/*z* 529.17044 (C_27_H_29_O_11_, error ≤ 2 ppm) in the positive and negative ion modes, respectively. They were correspondingly 176 Da smaller than **M6** and **M9**, that is to say, 176 Da larger than XN, from which the glucuronide acid (GluA) products of XN were deduced. In their ESI-MS^2^ spectra, the fragment ions at *m*/*z* 531 [M + H-GluA]^+^ and *m*/*z* 529 [M − H-GluA]^−^ confirmed our guess. Furthermore, a battery of characteristic fragment ions at *m*/*z* 233 and *m*/*z* 119 revealed the RDA reaction.

For metabolite **M11** with retention at 5.70 min, **M11** was detected at *m*/*z* 545.16645 in the mass spectrum, which increased by 192 Da compared with the size of XN. We deduced that it was a cyclization product with hydroxylation. In the secondary mass spectrum, **M11** possessed the main fragment ions at *m*/*z* 369, *m*/*z* 545, *m*/*z* 119, and *m*/*z* 249 and was yielded by a sequential drop of C_6_H_8_O_6_ and RDA cleavage, which supported our deduction.

Metabolites **M12**, **M20**, **M22**, **M25**, **M27**, and **M36** (C_27_H_29_O_12_, mass error < 5 ppm) possessed theoretical [M − H]^−^ at *m*/*z* 545.16535 and [M + H]^+^ at *m*/*z* 547.18100 with retention times of 5.72, 6.43, 6.43, 6.69, 6.80, and 8.39 min, respectively. In their MS/MS spectra, a neutral loss of 176 Da (*m*/*z* 545 → *m*/*z* 369) was observed, which indicated the occurrence of glucuronidation. Characteristic product ions at *m*/*z* 250 [M-H-C_8_H_8_O-GluA]^−^ and *m*/*z* 369 [C_21_H_22_O-H+O]^−^ were detected in the ESI-MS^2^ spectra. They were 16 Da larger than the corresponding ions of **M29**, **M31**, **M48**, and **M50**, which implied that they were identified as glucuronidation and hydroxylation metabolites.

**M3** (C_27_H_31_O_13,_ with mass error -3.826 ppm), 16 Da and 32 Da larger than **M12** and **M5**, respectively, was deduced from the oxidation products combined with two O on the basis of the glucuronide products of XN. The fragment ions at *m*/*z* 563 and *m*/*z* 385 [M + H-GluA]^+^ indicated the occurrence of glucuronidation and hydroxylation in the plasma of rats.

Eluted at 8.24 min and 9.71 min, **M34** and **M43** ([M + H]^+^/[M − H]^−^ molecular formula, C_26_H_29_O_11_ and C_26_H_27_O_11_) possessed the theoretical mass at *m*/*z* 515.15479 in [M − H]^−^ ion mode and at *m*/*z* 517.17044 in [M + H]^+^ ion mode, respectively, both of their errors <5 ppm. **M34** and **M43** were 14 Da (353 → 339) smaller than **M29**, **M31**, **M48**, and **M50**, which demonstrated that not only glucuronidation but also demethylation occurred.

#### 2.3.3. Identification of SMMC3

**M19**, **M21**, and **M44** afforded the [M − H]^−^ ion at *m*/*z* 609.12725 (C_27_H_29_O_14_S) with a mass error < 5.000 ppm. The DPI losses of 80 Da (609 → 529, 433 → 353) and 176 Da (609 → 433, 529 → 353) were observed in their ESI-MS/MS spectra. We inferred that they were the products of XN, successively through glucuronidation and sulfation. If both lost sulfate (80 Da) and glucuronic acid (176 Da) successively, it would ultimately become XN at *m*/*z* 353. As shown, the fragment ions at *m*/*z* 529, *m*/*z* 433, *m*/*z* 353, *m*/*z* 119, and *m*/*z* 233 provided a theoretical basis for our assertion, further confirming our inference. In our study, the metabolites were mainly observed in plasma.

**M4** was eluted at 5.07 min and showed deprotonated molecular ions at *m*/*z* 625.12217 (C_27_H_29_O_15_S, mass error of 1.412 ppm). The neutral losses of 80 Da (449 → 369) and 176 Da (625 → 449) indicated the presence of SO_3_ and GluA. Additionally, **M4** was 16 Da larger than **M19**, **M21**, and **M44**; thus, we deduced that **M4** was the oxidation metabolite of **M19**. The fragment ion at *m*/*z* 249 (16 Da higher than the RDA fragment at *m*/*z* 233) confirmed our deduction.

With a retention time of 7.71 min, **M32** displayed the deprotonated molecular ions (C_27_H_31_O_14_S) at *m*/*z* 611.1429 with a mass error of 0.470 ppm. In the ESI-MS^2^ spectrum, the DPIs at *m*/*z* 119 [C_8_H_8_O]^−^, *m*/*z* 353 [M-H-GluA-SO_3_H-2H]^−^, and *m*/*z* 531 [M-H-SO_3_H]^−^ were generated; thus, **M32** was tentatively characterized as hydrogenation products of **M19**.

#### 2.3.4. Identification of XN Metabolites except for SMMCs

**M33**, **M37**, and **M51**, three isomers, gave rise to their theoretical molecular [M + H]^+^ ion at *m*/*z* 369.13326 (C_21_H_21_O_6_, mass error within ± 3.00 ppm) with retention times of 8.07 min, 8.42 min, and 11.79 min, respectively. As we can see, at *m*/*z* 371 all three ions were detected, indicating two hydrogen atoms were added to them. They were 16 Da larger than XN, which indicated that oxidation occurred. The fragment ions at *m*/*z* 249 and *m*/*z* 119 were detected, suggesting that the oxidation took place on the A-ring instead of the B-ring. We tentatively deduced that oxidation might take place on the double bond or one of the two terminal methyl groups of the prenyl moiety. However, by comparing our data with that of the literature [15], we further confirmed that oxidation took place on the terminal methyl groups of the prenyl moiety. The fragment ion detected at *m*/*z* 339 corresponded to the loss of CH_2_O, which strongly supported our deduction. Hence, **M33**, **M37**, and **M51** were characterized as the oxidation products of XN.

The main relative intensity for the negative ion tandem mass spectrum of metabolite **M46** was 64.76% at *m*/*z* 119, indicating that the possible charging-carrying groups on the A-ring were blocked. Thus, it directed fragmentation exclusively to the B-ring. Fragment ions detected at *m*/*z* 235 confirmed our suggestion. Hence, **M46** was a cyclization product formed by an intramolecular attack of the 4′-hydroxyl group. An intramolecular reaction with the epoxide might be a detoxification pathway that prevents the epoxide from reacting with biological nucleophiles on important molecules such as proteins or nucleic acids [15].

With a retention time of 10.47 min, **M47**, 32 Da larger than XN, afforded the [M − H]^−^ ions at *m*/*z* 385.12875 (C_21_H_21_O_7_, mass error 1.482 ppm) in the negative ion mode. We deduced that **M47** was the oxidation product of XN. In the ESI-MS/MS spectrum, the appearance of an ion at *m*/*z* 265 confirmed our hypothesis, which occurred on the A-ring rather than the B-ring.

**M49** (C_21_H_23_O_8_), with an error −1.077 ppm, was eluted at 10.79 min. **M49**, possessing the [M + H]^+^ ions at *m*/*z* 403.13874, was 48 Da larger than XN, indicating the hydroxylation product of XN. In its ESI-MS^2^ spectrum, the DPIs at *m*/*z* 403 and at *m*/*z* 355 indicated the successive loss of hydroxy; therefore, **M49** was tentatively identified as an XN oxidation metabolite, but the exact active site could not be determined.

## 3. Discussion

### 3.1. Comparison of Three Different Biological Treatment Methods

**Figure 6:**
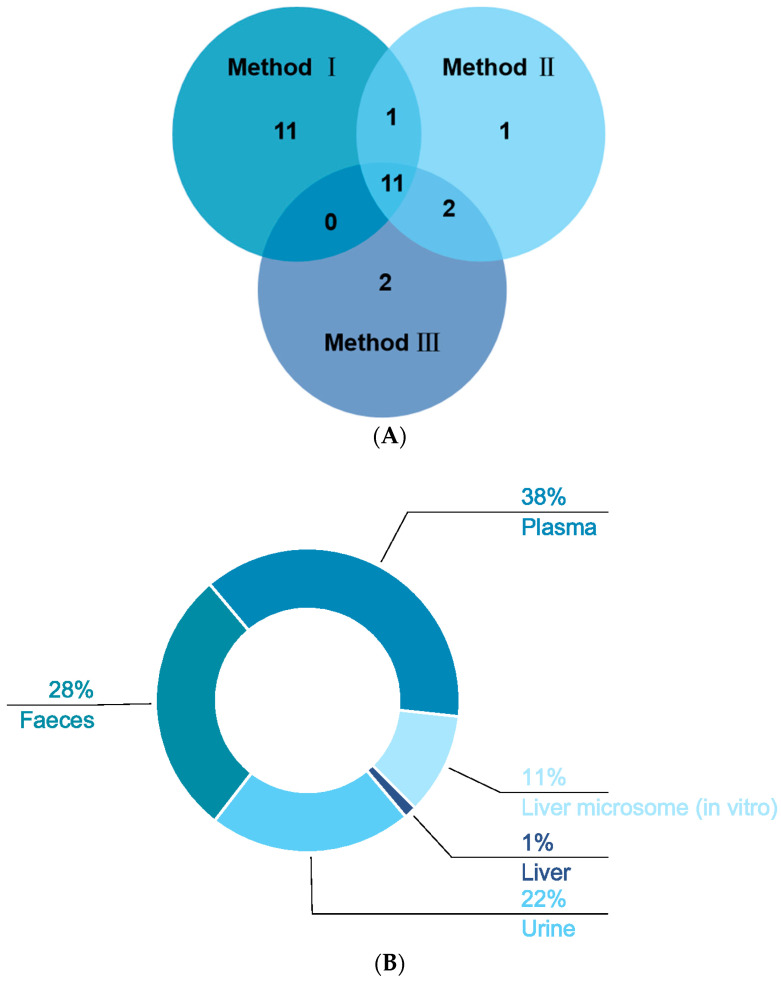
Distribution of XN metabolites under three different treatments (**A**) and the distribution of XN metabolites in vivo and in vitro (**B**).

In our study, three methods were used to pretreat the biological samples, and then we applied the same analytical method to detect the signals (see Supplementary Materials). A total of 53 XN metabolites from phases Ⅰ and Ⅱ were screened and identified in vivo and in vitro by using the UHPLC-Q-Exactive Orbitrap method, all of which were based on accurate mass measurements, fragmentation patterns, diagnostic product ions, and literature reports. Plasma samples were characterized as low in content, complex in matrix, and rich in protein; thus, pretreatment played an irreplaceable role in the sample processing. Consequently, protein removal was the top priority. Herein, 23 plasma metabolic products were acquired by SPE, named “method I”. Fifteen metabolites were, respectively, screened by methanol precipitation (method II) and acetonitrile precipitation (method III), as shown in Figure 6A. In general, the three methods mentioned above are widely applied to pretreat the samples in various laboratories at present. Plasma sample preparation approaches are often based on deproteinization using solvents [29] such as acetonitrile and methanol, but detector saturation and ion suppression caused by high concentrations of remaining proteins or species such as phospholipids (PLs) or lysophospholipids (LPLs) are often reported [30]. Meanwhile, we adopted the method of SPE to process biological samples, enriching the target substance and removing the impurities before instrumental analysis, aiming to improve analytical sensitivity and reduce damage to the instrument [31,32]. As we have seen, the number of metabolites by SPE was greater than that by methods Ⅱ and Ⅲ, which might indicate that the SPE cartridge could enrich these kinds of metabolites. Based on the results, it could be inferred that the majority of the metabolites were obtained by method I after the same detection analysis, which showed that the SPE cartridge was the optimal choice for plasma sample enrichment and purification. After the sample was concentrated by the SPE cartridge, more response signals could be detected in mass spectrometry, which provided a certain research basis for the further study of biological samples.

### 3.2. Comparison of XN Metabolites In Vivo and In Vitro

In our study, we identified one, eight, sixteen, twenty-one, and twenty-eight metabolites in liver (1%), liver microsomes (11%), urine (22%), feces (28%), and plasma (38%), respectively, of which eight in vitro and fifty-two in vivo (in Figure 6B). While there were interrelated phenomena, we still concluded that the metabolites in vivo were far more abundant than the metabolites in vitro. An in vitro metabolism study indicated that gluconuronidation and sulfation metabolites of XN were primarily metabolites, which was in accordance with the discovery by M. Yilmazer et al. [13]. As a result, these metabolites in vivo were presumed to be generated through sulfation, glucuronidation, dehydrogenation, methylation, hydrogenation, hydroxylation, ring cleavage, and their composite reactions. In M. Yilmazer’s study, based on other flavonoids such as diosmetin, quercetin, genistein, rutin, and kaempferol, they first demonstrated that XN, a prenylated flavonoid, produced glucuronides by liver microsomes from humans or rats using HPLC, UV spectroscopy, liquid chromatography/mass spectrometry (LC/MS), and proton nuclear magnetic resonance (^1^H-NMR). In our recent study, in addition to the glucuronidation reaction, oxidation and hydroxylation reactions took place. In vivo metabolism: metabolites in plasma were a few more than in feces and urine via method Ⅰ; nevertheless, there was merely one metabolite in the liver. Furthermore, **M37**, **M46**, **M47**, **M48**, and **M49** were detected in vitro; that is to say, oxidation, epoxidation of the prenyl group, and glucuronidation occurred. At the same time, we found that the prenyl group in the A ring of prenylchalcones is a major site for hepatic metabolism [14].

We can infer that the metabolic pathways of XN are extensive, indicating that XN has a larger window. On the whole, metabolites exist in the form of multiple metabolites; glucaldehydes acidification and sulfate esterification are at most followed by hydroxylation, hydrogenation, oxidation, and so on. Glucuronidation, considered a detoxification process or a defense mechanism that helps the body remove unwanted substances, including endogenous substances, attaches a glucuronide moiety to a substrate, making a product that is highly hydrophilic. Moreover, after oral administration of botanical dietary supplements produced from hops (*Humulus lupulus*) containing the chemopreventive compound XN, it has been reported that abundant diglucuronides and sulfate-glucuronic acid diconjugates were observed in the metabolites of perimenopausal and postmenopausal women, which were used by women to manage menopausal symptoms [33]. The glucuronides are then often eliminated via bile or urine. Hence, glucuronidation is an essential biological process in humans, protecting the body from excessive accumulation of toxic substances. Glucuronidation serves as the primary elimination pathway for a variety of drugs on the market [34].

### 3.3. Comparison of Metabolites Identified in This Study and in Previous Studies

Various phase I metabolites formed by dehydrogenation, demethylation, dehydration, hydroxylation, or epoxidation reactions have been described. Concerning phase II metabolites, glucuronidation and sulfation have also been reported in the literature for in vitro experiments [10,13]. Robert J. et al. described the oxidation, demethylation, hydration, and sulfation reactions in rat feces via high-mass accuracy HPLC/ESI-MS/MS measurements; however, no glucuronide was found, contrary to the conclusions stated in [35]. Few studies reported metabolites in both phases I and Ⅱ comprehensively at the same time. Only six metabolites of XN were identified in the plasma, urine, and feces samples of rats by Bai et al. [36], including methylated, glucuronidated, acid-catalyzed cyclization, and oxidation. However, in our study, we identified 53 XN metabolites, of which some simultaneously possess two glucuronides-bounding or glucuronides and sulfating-bounding, which indicated our method was superior to the method that has been reported.

## 4. Materials and Methods

### 4.1. Chemicals and Reagents

XN was purchased from Chengdu Must Biotechnology Co., Ltd. (Sichuan, China). The reference standard with a purity higher than 98% was applicable to HPLC-UV analysis. HPLC-grade acetonitrile, methanol, and formic acid (FA) were purchased from Thermo Fisher Scientific (Fair Lawn, NJ, USA). All the other chemicals of analytical grade were available at the workstation, Beijing Chemical Works (Beijing, China). Deionized water used throughout the experiment was purified by a Milli-Q Gradient Å 10 System (Millipore, Billerica, MA, USA). Grace Pure SPE C_18_-Low solid-phase extraction cartridges (200 mg/3 mL and 59 μm, 70 Å) were purchased from Grace Davison Discovery Science (Deerfield, IL, USA).

### 4.2. Animals and Drug Administration

Six male SD rats weighing 200 ± 10 g were obtained from the Jinan Pengyue Experimental Animals Company (Jinan, China). The rats were housed in a controlled room at standard temperature (24 ± 2 °C) and relative humidity (70 ± 5%) with a 12 h light/12 h dark cycle in Shandong International Biotechnology Park. After a week of acclimation, the rats were randomly divided into two groups: the drug group (*n* = 3) for test plasma, urine, feces and liver tissues, and the control group (*n* = 3) for blank plasma, urine, feces, and liver tissues. The rats were fasted for 12 h with free access to water prior to the experiment. XN was suspended in physiological saline. Rats in the drug group were given a dose of 150 mg/kg body weight orally. Physiological saline (2 mL) was parallelly administrated to rats in the control group. All animal experiments were performed according to a protocol approved by the Institutional Animal Care and Use Committee at Bin Zhou Medical University (Lot:2021-083). The animal facilities and protocols complied with the Guide for the Care and Use of Laboratory Animals (USA National Research Council, 1996).

### 4.3. Sample Collection and Pretreatment In Vivo

#### 4.3.1. Sample Collection

For plasma sample collection, each blood sample (500 μL) was gathered from the suborbital venous plexus of rats at 0.5, 1.0, 1.5, 2.0, 4.0, and 6.0 h after the final dosing, respectively, and the plasma samples were collected into heparinized glass tubes. The supernatant was later obtained by centrifuging the blood at 3500 r/min for 10 min, and the samples were then merged and frozen at −80 °C. All the samples were divided evenly into three groups.

For tissue sample collection, rats were dissected, and liver tissue samples were collected after oral administration for 24 h. At the end of the administration, two groups of rats were sacrificed in parallel. Liver tissue was obtained, quenched in liquid nitrogen, and then stored at −80 °C.

For urine and feces collection, two groups of rats were housed in metabolism cages with free access to water but a ban on food until the experiment. The urine and fecal samples were gathered at 0–24 h after oral administration and then merged separately. The urine samples were kept in the −80 °C refrigerator after centrifuging at 12,000 r/min for 15 min, whereas the fecal samples were collected after freeze-drying and ground into powder, which was frozen in a centrifuge tube. The resulting urine and fecal samples were merged and stored frozen (−80 °C) until analysis.

All homogeneous biological samples from the same group were finally merged into a collective sample.

#### 4.3.2. Sample Pretreatment

The solid phase extraction (SPE) technology, a method for precipitation and concentration of protein and solid residues, was applied to pretreat all the biological samples [31,37,38].

One of the three merged plasma samples (1 mL) was added to SPE cartridges pretreated following methanol (3 mL) and deionized water (3 mL), respectively. Then, the SPE cartridges were successively washed with deionized water (3 mL) and methanol (3 mL). The methanol eluent was collected and dried under N_2_ at room temperature. The residue was then redissolved in 300 µL of methanol and centrifuged for 30 min (14,000 r/min, 4 °C). The obtained supernatant was used for the subsequent analysis. Moreover, the other two plasma samples were mixed with three times the volume of acetonitrile and methanol, respectively. The resulting mixture was centrifuged again at 3500 r/min for 20 min, and the supernatant was dried under N_2_. After drying, the residue was dried with 200 μL of a 1:1 (*v*/*v*) acetonitrile-water solution, vortexed, and then centrifuged at 14,000 r/min for 30 min. The supernatant was transferred to an injection bottle cannula for UHPLC-MS/MS analysis.

The merged urine samples (1 mL) were centrifuged at 12,000 r/min for 30 min for the purpose of supernatant collection. Feces samples (2 g) were ultrasonically dissolved with deionized water (10 mL) for 30 min. The supernatant was acquired, followed by the mixture being centrifuged at 3500 r/min for 20 min. Moreover, liver tissue was dissolved in 10 mL of normal saline after being ground, and the supernatant (1 mL) was centrifuged at 3500 r/min for 20 min. Then, the subsequent treatments performed were prepared by solid phase extraction (SPE). Firstly, the SPE column was prepared with methanol (3 mL) to activate it, followed by water (3 mL). Subsequently, urine, feces, and liver tissue samples (1 mL) were added to the pretreated SPE column and subsequently washed with water (3 mL) and methanol (3 mL). Ultimately, the methanol eluate was finally collected into a 5 mL EP tube and evaporated by nitrogen at room temperature.

All the supernatants were used for further instrumental analysis.

### 4.4. Sample Collection and Pretreatment In Vitro

#### 4.4.1. In Vitro Metabolism Incubation

A typical biotransformation incubation mixture consists of 1 mg/mL microsome protein in phosphate buffer saline (PBS), pH 7.4, containing 3 mM MgCl_2_, and 0.1 mg/mL XN (dissolved in methanol, concentration < 1%) as substrate. Control incubations were performed without the addition of uridine diphosphate glucuronic acid (UDPGA) or microsomes. The reaction was initiated by the addition of 100 μL of 25 mg/mL NADPH after 5 min of preincubation of the substrate and the microsome protein at 37 °C. Incubations were carried out at 37 °C continuously until the reaction was terminated by adding 200 μL of ice-cold acetonitrile to a 100 μL compound system solution after 5, 10, 15, 30, 45, 60, 120, and 240 min, respectively.

#### 4.4.2. Sample Pretreatment

The samples were gathered at 0–240 min and merged separately. The supernatant mixture was then centrifuged at 3500 r/min for 15 min, and the subsequent treatments performed were the same as those in the plasma samples. All the supernatants were used for further instrumental analysis.

### 4.5. Instruments and Analytical Conditions

#### 4.5.1. UHPLC Parameters

Chromatographic separation was carried out on a Dionex Ultimate 3000 UHPLC system (Thermo Fisher Scientific, Waltham, MA, USA). Separation was performed on a Waters ACQUITY BEH C_18_ column (2.1 × 100 mm, 1.7 μm). A flow rate of 0.30 mL/min was set for the separation of the target metabolites. The column temperature was maintained at 30 °C, and the injection volume was 3 μL. The mobile phase was composed of acetonitrile (A) and water containing 0.1% FA (B). The gradient elution conditions were set as follows: 0.0–5.0 min, 95.0% B; 5.0–10.0 min, 95–70% B; 10.0–27.0 min, 70–50% B; and 27.0–27.1 min B, 95–10%; 27.1–30.0, 10–95% B.

#### 4.5.2. HRMS Parameters

All the LC-MS analyses were performed on a Q-Exactive Orbitrap Mass spectrometer (Thermo Electron, Bremen, Germany) via a heated electrospray ionization (HESI) source. Mass spectrometric detection was performed in positive and negative ion modes in a single analytical run. The ion source parameters were listed as follows: nitrogen (purity ≥ 99.99%) served as the sheath gas and auxiliary gas at a flow rate of 45 and 10 (arbitrary units), respectively; a capillary temperature of 320 °C, a vaporizer temperature of 320 °C, and a spray voltage of 3800/3500 V (+/−) were used. A high-resolution mass spectrum was acquired at full scan in a mass range of *m*/*z* 80–1200 at a resolution of 70,000 detected by an Orbitrap analysis. The ESI-MS/MS data at a resolution of 35,000 were obtained by a parallel reaction monitoring mode triggered by an inclusion ion list, which was built by molecular prediction. The collision energy was set at the normalized collision energy of 30% to generate the fragment ions.

### 4.6. Peak Selections and Data Processing

The Thermo Xcalibur 2.1 workstation was used for data acquisition and processing. To obtain as many ESI-MS/MS fragment ions of XN metabolites as possible, the peaks detected with intensities over 10,000 were selected for identification. The accurate mass of chemical formulas attributed to all parent ions of the selected peaks was calculated using a formula predictor by setting the parameters as follows: C [0–35], H [0–40], O [0–16], and S [0–5]. The ring double bond (RDB) equivalent value [0–15] and accurate mass measurements were set within a mass error of ±5 ppm.

## 5. Conclusions

In the present study, the in vivo and in vitro metabolic profiles of XN were thoroughly investigated using UHPLC-Q-Exactive Orbitrap mass spectrometry, combining standard substances, fragmentation patterns in the literature, chromatographic behaviors, and relevant drug biotransformation knowledge coupled with offline data processing methods. A total of 53 metabolites were thoroughly screened and identified in the plasma, urine, feces, liver, and liver microsomes of rats after XN oral administration. The major phase I metabolic pathways of XN in mice involved hydroxylation, hydrolysis, hydrogenation, oxidation, and ketone formation, while those of phase II reactions primarily included glucuronidation and sulfation. The unique metabolic reaction of sulfation was detected in rat feces, and glucuronidation was only detected in plasma and urine, indicating that feces, plasma, and urine samples would be more appropriate for investigating the conjugation reaction of phase II metabolism. More importantly, this work illustrated a stepwise targeted matching strategy, based on the SMM and SMMC, for rapid and effective characterization of XN metabolites, which was of significance to the metabolite profile of other prenylated flavonoid compounds; however, as to TCMs (traditional Chinese medicines), how to confirm the SMM is now pretty much the agenda for this strategy.

## Figures and Tables

**Table 1:** Summary of XN metabolites in rat urine, plasma, feces, and liver and in liver microsomes.

Peak	tR /min	Ion Mode	Formula [M − H]^−^/[M − H]^+^	Theoretical Mass (*m*/*z*)	Experimental Mass (*m*/*z*)	RDB	Error (ppm)	MS/MS Fragment Ions	P	F	G	GW	U
M0 *	14.69	P	C_21_H_23_O_5_	355.15400	355.15323	10.5	−2.169	179 (100.00), 299 (27.29), 355 (14.12), 197 (7.75), 356 (3.29), 235 (0.84), 233 (0.31), 357 (0.26), 353 (0.14)					
	14.69	N	C_21_H_21_O_5_	353.13944	353.13861	11.5	0.736	119 (100.00), 353 (78.54), 233 (30.19), 354 (18.16), 120 (8.03), 234 (4.14), 145 (2.52), 247 (2.00), 283 (1.93), 355 (1.62), 159 (1.45)	√	√		√	√
M1	1.42	N	C_22_H_23_O_9_S	463.10573	463.10278	11.5	−6.369	96 (100.00), 463 (5.63), 365 (4.79), 245 (2.98), 299 (1.14)					√
M2	4.83	N	C_21_H_23_O_10_S	467.10064	467.10120	10.5	1.190	119 (100.00), 385 (48.67), 467 (10.81)		√			
M3	4.88	P	C_27_H_31_O_13_	563.17592	563.17151	12.5	−7.826	563 (16.20), 385 (3.61), 366 (8.21)	√				
M4	5.07	N	C_27_H_29_O_15_S	625.12217	625.12305	13.5	1.412	449 (100.00), 369 (75.22), 119 (64.83), 249 (57.81), 625 (21.82), 545 (8.01)	√				
M5	5.1	P	C_27_H_31_O_11_	531.18609	531.18573	12.5	−0.674	179 (100.00), 299 (32.16), 531 (14.45), 355 (9.42), 235 (1.19)					√
M6	5.11	N	C_33_H_37_O_17_	705.20253	705.20361	15.5	−0.018	529 (100.00), 119 (51.21), 353 (48.85), 233 (32.89), 705 (23.70)	√				√
	5.12	P	C_33_H_39_O_17_	707.21818	707.21741	14.5	−1.083	179 (100.00), 299 (28.94), 531 (16.52), 355 (8.21), 235 (1.26), 707 (0.98)					√
M7	5.11	N	C_21_H_21_O_12_S	497.07482	497.07599	11.5	2.347	497 (35.08), 322 (16.38), 119 (5.20), 417 (4.32)	√				
M8	5.5	N	C_21_H_21_O_12_S	497.07482	497.07578	11.5	1.925	497 (44.86), 241 (23.40), 322 (17.93), 417 (7.09), 119 (5.27)	√				
M9	5.59	P	C_33_H_39_O_17_	707.21818	707.21771	14.5	−0.659	179 (100.00), 531 (46.80), 299 (17.24), 355 (15.10), 707 (9.62), 235 (5.66)	√				
	5.6	N	C_33_H_37_O_17_	705.20253	705.20349	15.5	1.367	529 (100.00), 353 (39.87), 705 (34.73), 119 (33.47), 233 (31.31)	√				√
M10	5.61	N	C_21_H_23_O_10_S	467.10064	467.10101	10.5	0.784	119 (100.00), 387 (35.11), 467 (11.12), 265 (4.95)		√			
M11	5.70	N	C_27_H_29_O_12_	545.16645	545.16650	13.5	2.105	369 (100.00), 119 (77.41), 249 (56.43), 545 (51.02)	√				
	5.72	P	C_27_H_31_O_12_	547.17914	547.18100	12.5	−1.394	371 (30.35), 547 (4.33), 355 (1.51)	√				
M12	5.72	N	C_27_H_29_O_12_	545.16535	545.16675	13.5	2.563	369 (100.00), 119 (77.41), 545 (51.02), 250 (7.31), 339 (2.73)	√				
	5.72	P	C_27_H_31_O_12_	547.18100	547.18091	12.5	−0.169	371 (30.35), 547 (4.33), 355 (1.51), 235 (0.94), 179 (0.53)	√				
M13	5.96	N	C_21_H_23_O_10_S	467.10064	467.10117	10.5	1.126	119 (100.00), 387 (19.87), 249 (19.40), 467 (17.71)		√			
M14	6.15	N	C_21_H_23_O_9_S	451.10573	451.10580	10.5	0.157	119 (100.00), 369 (47.42), 451 (10.37), 119 (0.62), 249 (0.39)	√				
M15	6.28	N	C_21_H_23_O_9_S	451.10573	451.10577	10.5	0.090	119 (100.00), 371 (49.53), 249 (28.42), 451 (12.79)		√			
M16	6.32	N	C_21_H_23_O_10_S	467.10064	467.10071	10.5	0.141	385 (100.00), 119 (71.51), 467 (43.08), 265 (2.28), 249 (1.60)		√			
M17	6.36	N	C_21_H_21_O_10_S	465.08499	465.08456	11.5	−3.291	119 (91.97), 385 (44.65), 465 (26.62)		√			
M18	6.39	P	C_21_H_27_O_9_S	455.13703	455.13586	8.5	−2.570	179 (72.17), 251 (25.44), 235 (22.41), 455 (0.58), 375 (0.26)		√			
M19	6.42	N	C_27_H_29_O_14_S	609.12725	609.12848	13.5	2.015	433 (100.00), 353 (69.97), 233 (50.44), 119 (49.39), 609 (19.48), 529 (7.03)	√				
M20	6.43	N	C_27_H_29_O_12_	545.16535	545.16620	13.5	1.554	369 (10000), 545 (6.99), 233 (4.28)	√				
M21	6.43	N	C_27_H_29_O_14_S	609.12725	609.12817	13.5	1.506	433 (100.00), 353 (78.90), 119 (53.13), 233 (53.04), 609 (20.17)	√				
M22	6.43	N	C_27_H_29_O_12_	545.16535	545.16602	13.5	−0.788	369 (100.00), 545 (12.71), 247 (8.36)	√				√
	6.44	P	C_27_H_31_O_12_	547.18100	547.18054	12.5	−0.846	371 (100.00), 179 (24.36), 353 (10.51), 547 (7.77), 235 (3.87)	√				
M23	6.5	P	C_27_H_31_O_14_S	611.14290	611.14233	12.5	−0.937	179 (100.00), 435 (92.64), 299 (50.08), 355 (44.78), 235.10 (19.05), 531 (5.74), 611 (5.71)	√				
M24	6.64	N	C_21_H_21_O_10_S	465.08499	465.08536	11.5	−1.571	119 (100.00), 385 (21.03), 465 (19.85)		√			
M25	6.69	N	C_27_H_29_O_12_	545.16535	545.16595	13.5	−0.916	119 (100.00), 545 (42.66), 369 (17.50)					√
M26	6.77	N	C_21_H_21_O_9_S	449.09008	449.09015	11.5	0.071	119 (100.00), 369 (13.76), 449 (10.04), 233 (0.31), 96 (1.58)		√			
M27	6.86	N	C_27_H_29_O_12_	545.16535	545.16614	13.5	−0.568	119 (100.00), 545 (67.44), 369 (29.05), 247 (10.98)					√
M28	6.9	N	C_21_H_23_O_9_S	451.10573	451.10596	10.5	0.511	369 (100.00), 119 (70.53), 249 (40.01), 451 (14.02)	√				
M29	7.24	N	C_27_H_29_O_11_	529.17044	529.17096	13.5	0.986	119 (100.00), 353 (99.38), 233 (63.30), 529 (59.40)	√				√
	7.24	P	C_27_H_31_O_11_	531.18609	531.18549	12.5	−1.126	179 (100.00), 355 (62.21), 299 (19.94), 531 (5.15)	√				√
M30	7.36	N	C_27_H_27_O_12_	543.1497	543.150270	14.5	−0.975	367 (100.00), 543 (12.03), 281 (3.18)	√				
M31	7.49	P	C_27_H_31_O_11_	531.18609	531.18579	12.5	−0.561	179 (100.00), 531 (34.67), 355 (21.47), 299 (12.67)	√				√
	7.51	N	C_27_H_29_O_11_	529.17044	529.17096	13.5	0.986	529 (100.00), 119 (52.89), 233 (40.54), 353 (23.06)					√
M32	7.71	N	C_27_H_31_O_14_S	611.14290	611.14319	12.5	0.470	119 (100.00), 353 (71.68), 611 (60.17), 369 (4.25), 531 (1.51)		√			
M33	8.07	P	C_21_H_21_O_6_	369.13326	369.13251	11.5	−2.045	369 (100.00), 249 (15.06), 179 (4.16), 85 (3.22)				√	√
M34	8.24	N	C_26_H_27_O_11_	515.15479	515.15570	13.5	−0.359	339 (100.00), 515 (34.79), 119 (32.87)	√				
	8.24	P	C_26_H_29_O_11_	517.17044	517.17041	12.5	−2.175	341 (100.00), 517 (4.08), 179 (3.34)	√				
M35	8.30	N	C_21_H_21_O_8_S	433.09626	433.09540	11.5	0.543	119 (100.00), 233 (67.75), 353 (10.54), 433 (76.72),		√			√
	8.33	P	C_21_H_23_O_8_S	435.11191	435.10977	10.5	−2.401	179 (100.00), 435 (45.03), 355 (28.23), 299 (22.31), 235 (12.61), 121 (1.29),		√			
M36	8.39	N	C_27_H_29_O_12_	545.16535	545.16632	13.5	1.774	369 (100.00), 119 (75.73), 545 (32.54), 250 (5.78), 233 (1.51)	√				
	8.39	P	C_27_H_31_O_12_	547.18100	547.18091	12.5	−0.279	371 (32.58), 547 (3.94), 179 (3.21), 355 (1.47), 235 (0.96), 299 (0.61)	√				
M37	8.42	P	C_21_H_21_O_6_	369.13326	369.1322	11.5	−2.885	249 (100.00), 369 (51.07), 353 (9.42), 231 (6.52)		√		√	
M38	8.47	N	C_21_H_21_O_8_S	433.09626	433.09601	11.5	1.952	119 (100.00), 433 (83.63), 233 (71.85), 353 (58.14)					√
	8.47	P	C_21_H_23_O_8_S	435.11191	435.1102	10.5	−1.413	179 (100.00), 435 (47.52), 355 (26.88), 299 (20.88), 235 (11.17), 121 (0.75)					√
M39	8.65	N	C_21_H_21_O_9_S	449.09058	449.09008	11.5	1.115	119 (100.00), 369 (83.93), 449 (46.95), 80 (34.52), 249 (36.97)		√			
M40	8.83	N	C_21_H_23_O_8_S	435.11081	435.11063	10.5	−0.425	353 (100.00), 119 (78.71), 233 (43.17), 435 (9.49),		√			
	8.86	P	C_21_H_25_O_8_S	437.12646	437.12494	9.5	−3.488	179 (100.00), 235 (43.37), 299 (17.34), 437 (6.22), 355 (1.60)		√			
M41	8.87	N	C_21_H_23_O_10_S	467.10064	467.10071	10.5	0.1410	119 (35.08), 467 (6.44), 387 (4.67), 247 (2.51)		√			
M42	9.28	N	C_21_H_23_O_8_S	435.11081	435.11194	10.5	2.586	353 (100.00), 119 (68.81), 233 (35.24), 435 (11.67), 96 (2.77)	√				
	9.30	P	C_21_H_25_O_8_S	437.12646	437.12604	9.5	−0.972	179 (100.00), 235 (44.98), 299 (11.31), 437 (9.22)					√
M43	9.71	N	C_26_H_27_O_11_	515.15479	515.15521	13.5	−1.310	339 (100.00), 515 (30.45), 119 (27.85)	√				
	9.71	P	C_26_H_29_O_11_	517.17044	517.16968	12.5	−3.587	285 (100.00), 165 (67.89), 341 (18.93), 517 (12.74)	√				
M44	9.99	N	C_27_H_29_O_14_S	609.12725	609.12817	13.5	1.506	609 (100.00), 353 (86.40), 119 (66.91), 233 (63.16), 529 (29.62), 433 (13.42)	√				
M45	10.03	N	C_21_H_19_O_9_S	447.07443	447.0752	12.5	1.724	119 (100.00), 447 (23.81), 367 (22.27), 96 (1.24), 351 (0.18)		√			
M46	10.37	P	C_21_H_23_O_6_	371.14891	371.14767	10.5	−0.013	371 (100.00), 179 (24.36), 353 (10.51), 547 (7.77), 235 (3.87)	√	√		√	√
	10.36	N	C_21_H_21_O_6_	369.13333	369.13436	11.5	1.504	369 (100.00), 119 (93.76), 250 (3.65), 339 (2.56)	√	√	√	√	√
M47	10.47	N	C_21_H_21_O_7_	385.12818	385.12875	11.5	1.482	385 (100.00), 119 (64.76), 265 (1.34), 149 (1.08)				√	
M48	10.63	N	C_27_H_29_O_11_	529.17044	529.17108	13.5	1.213	353 (100.00), 119 (87.24), 233 (53.66), 529 (35.90)	√			√	√
	10.63	P	C_27_H_31_O_11_	531.18609	531.18542	12.5	−1.258	179 (100.00), 299 (32.08), 531 (12.19), 355 (8.72), 235 (1.11)	√				√
M49	10.79	P	C_21_H_23_O_8_	403.13874	403.13831	10.5	−1.077	403 (75.61), 355 (5.95), 179 (1.20), 299 (1.06)				√	
M50	11.45	N	C_27_H_29_O_11_	529.17044	529.17108	13.5	1.213	119 (95.09), 353 (67.23), 233 (54.97), 529 (41.65)	√				
	11.46	P	C_27_H_31_O_11_	531.18609	531.18542	12.5	−1.258	179 (100.00), 531 (14.03), 299 (4.75), 355 (4.00), 235 (1.33)	√				
M51	11.79	P	C_21_H_21_O_6_	369.13326	369.13293	11.5	−0.907	249 (100.00), 369 (78.55), 353 (31.27), 231 (20.32), 351 (4.32)		√		√	
M52	13.08	P	C_21_H_23_O_8_S	435.11191	435.10992	10.5	−2.056	179 (100.00), 435 (12.91), 299 (1.82), 355 (1.14), 235 (0.61)		√			
	13.09	N	C_21_H_21_O_8_S	433.09626	433.09583	11.5	1.536	119 (100.00), 353 (65.57), 433 (64.65), 233 (63.70)	√				
M53	24.15	N	C_28_H_31_O_13_	575.17592	575.17914	13.5	5.603	257 (73.88), 433 (24.22), 233 (23.17), 119 (15.49), 575 (4.19)		√			

Note: tR: retention time; P: positive ion mode; N: negative ion mode; *: standard substance; U: urine; P: plasma; F: feces; G: liver; GW: liver microsome; and “√”: detected.

## Data Availability

Most of the data used during the preparation of the manuscript are included in the Results and Discussion sections. However, for any additional details of the procedures and the original raw files, please contact the corresponding authors.

## Supplementary Materials

The following supporting information can be downloaded at: https://www.mdpi.com/article/10.3390/molecules28135168/s1, Table S1: Summary of plasma metabolites pretreated by three different methods.

## Sample Availability

Samples of the related compounds are available from the authors.
