# Supplementary material for: Stepwise Targeted Matching Strategy for Comprehensive Profiling of Xanthohumol Metabolites In Vivo and In Vitro Using UHPLC-Q-Exactive Orbitrap Mass Spectrometer
Source: Molecules. 2023 Jul 2;28(13):5168. doi: 10.3390/molecules28135168 (PMC10343281; doi:10.3390/molecules28135168)
Supplement: Supplementary file 1 [file molecules-28-05168-s001.zip › molecules-2441766-supplementary.pdf]

Supplementary material:

Table S1: Summary of plasma metabolites pretreated by three different methods.

Peak	tR /min	Ion Mode	Formula [M-H]-/[M-H]+	Theoretical Mass (*m/z*)	Experimental Mass (*m/z*)	RDB	Error (ppm)	MS/MS fragment ions	Method		
									I	II	III
**M0***	14.69	P	$C_{21}H_{23}O_5$	355.15400	355.15323	10.5	-2.169	179(100.00), 299(27.29), 355(14.12), 197(7.75), 356(3.29), 235(0.84), 233(0.31), 357(0.26), 353(0.14)			
	14.69	N	$C_2H_2O_5$	353.13944	353.13861	11.5	0.736	119(100.00), 353(78.54), 233(30.19), 354(18.16), 120(8.03), 234.08(4.14), 145(2.52), 247(2.00), 283(1.93), 355(1.62), 159(1.45)	√	√	
**M1**	1.42	N	$C_{22}H_{23}O_9S$	463.10573	463.10278	11.5	-6.369	96(100.00), 463(5.63), 365(4.79), 245(2.98), 299(1.14)			
**M2**	4.83	N	$C_{21}H_{23}O_{10}S$	467.10064	467.10120	10.5	1.190	119(100.00), 385(48.67), 467(10.81)			
**M3**	4.88	P	$C_{27}H_{31}O_{13}$	563.17592	563.17151	12.5	-7.826	563(16.20), 385(3.61), 366(8.21)	√		
**M4**	5.07	N	$C_{27}H_{29}O_{15}S$	625.12217	625.12305	13.5	1.412	449(100.00), 369(75.22), 119(64.83), 249(57.81), 625(21.82), 545(8.01)	√		
**M5**	5.1	P	$C_{27}H_{31}O_{11}$	531.18609	531.18573	12.5	-0.674	179(100.00), 299(32.16), 531(14.45), 355(9.42), 235(1.19)			
**M6**	5.11	N	$C_{33}H_{37}O_{17}$	705.20253	705.20361	15.5	-0.018	529(100.00), 119(51.21), 353(48.85), 233(32.89), 705(23.70)	√		
	5.12	P	$C_{33}H_{39}O_{17}$	707.21818	707.21741	14.5	-1.083	179(100.00), 299(28.94), 531(16.52), 355(8.21), 235(1.26), 707(0.98)			
**M7**	5.11	N	$C_{21}H_{21}O_{12}S$	497.07482	497.07599	11.5	2.347	497(35.08), 322(16.38), 119(5.20), 417(4.32)		√	
**M8**	5.5	N	$C_{21}H_{21}O_{12}S$	497.07482	497.07578	11.5	1.925	497(44.86), 241(23.40), 322(17.93), 417(7.09), 119(5.27)		√	√
**M9**	5.59	P	$C_{33}H_{39}O_{17}$	707.21818	707.21771	14.5	-0.659	179(100.00), 531(46.80), 299(17.24), 355(15.10), 707(9.62), 235(5.66)	√	√	√
	5.6	N	$C_{33}H_{37}O_{17}$	705.20253	705.20349	15.5	1.367	529(100.00), 353(39.87), 705(34.73), 119(33.47), 233(31.31)	√	√	
**M10**	5.61	N	$C_{21}H_{23}O_{10}S$	467.10064	467.10101	10.5	0.784	119(100.00), 387(35.11), 467(11.12), 265(4.95)			
**M11**	5.70	N	$C_{27}H_{29}O_{12}$	545.16645	545.16650	13.5	2.105	369(100.00), 119(77.41), 249(56.43), 545(51.02)	√	√	√
	5.72	P	$C_{27}H_{31}O_{12}$	547.17914	547.18100	12.5	-1.394	371(30.35), 547(4.33), 355(1.51)	√	√	√
**M12**	5.72	N	$C_{27}H_{29}O_{12}$	545.16535	545.16675	13.5	2.563	369(100.00), 119(77.41), 545(51.02), 250(7.31), 339(2.73)	√		√
	5.72	P	$C_{27}H_{31}O_{12}$	547.18100	547.18091	12.5	-0.169	371(30.35), 547(4.33), 355(1.51), 235(0.94), 179(0.53)	√	√	
**M13**	5.96	N	$C_{21}H_{23}O_{10}S$	467.10064	467.10117	10.5	1.126	119(100.00), 387(19.87), 249(19.40), 467(17.71)			
**M14**	6.15	N	$C_{21}H_{23}O_9S$	451.10573	451.10580	10.5	0.157	119(100.00), 369(47.42), 451(10.37), 119(0.62), 249(0.39)			√
**M15**	6.28	N	$C_{21}H_{23}O_9S$	451.10573	451.10577	10.5	0.090	119(100.00), 371(49.53), 249(28.42), 451(12.79)			
**M16**	6.32	N	$C_{21}H_{23}O_{10}S$	467.10064	467.10071	10.5	0.141	385(100.00), 119(71.51), 467(43.08), 265(2.28), 249(1.60)			
**M17**	6.36	N	$C_{21}H_{21}O_{10}S$	465.08499	465.08456	11.5	-3.291	119(91.97), 385(44.65), 465(26.62)			
**M18**	6.39	P	$C_{21}H_{27}O_9S$	455.13703	455.13586	8.5	-2.570	179(72.17), 251(25.44), 235(22.41), 455(0.58), 375(0.26)			

**M19**	6.42	N	$C_{27}H_{29}O_{14}S$	609.12725	609.12848	13.5	2.015	433(100.00), 353(69.97), 233(50.44), 119(49.39), 609(19.48), 529(7.03)	√	√	√
**M20**	6.43	N	$C_{27}H_{29}O_{12}$	545.16535	545.16620	13.5	1.554	369(10000), 545(6.99), 233(4.28)	√		
**M21**	6.43	N	$C_{27}H_{29}O_{14}S$	609.12725	609.12817	13.5	1.506	433(100.00), 353(78.90), 119(53.13), 233(53.04), 609(20.17)	√	√	√
**M22**	6.43	N	$C_{27}H_{29}O_{12}$	545.16535	545.16602	13.5	-0.788	369(100.00), 545(12.71), 247(8.36)	√		
	6.44	P	$C_{27}H_{31}O_{12}$	547.18100	547.18054	12.5	-0.846	371(100.00), 179(24.36), 353(10.51), 547(7.77), 235(3.87)	√		
**M23**	6.5	P	$C_{27}H_{31}O_{14}S$	611.14290	611.14233	12.5	-0.937	179(100.00), 435(92.64), 299(50.08), 355(44.78), 235.10(19.05), 531(5.74), 611(5.71)	√	√	√
**M24**	6.64	N	$C_{21}H_{21}O_{10}S$	465.08499	465.08536	11.5	-1.571	119(100.00), 385(21.03), 465(19.85)			
**M25**	6.69	N	$C_{27}H_{29}O_{12}$	545.16535	545.16595	13.5	-0.916	119(100.00), 545(42.66), 369(17.50)			
**M26**	6.77	N	$C_{21}H_{21}O_{9}S$	449.09008	449.09015	11.5	0.071	119(100.00), 369(13.76), 449(10.04), 233(0.31), 96(1.58)			
**M27**	6.86	N	$C_{27}H_{29}O_{12}$	545.16535	545.16614	13.5	-0.568	119(100.00), 545(67.44), 369(29.05), 247(10.98)			
**M28**	6.9	N	$C_{21}H_{23}O_{9}S$	451.10573	451.10596	10.5	0.511	369(100.00), 119(70.53), 249(40.01), 451(14.02)			√
**M29**	7.24	N	$C_{27}H_{29}O_{11}$	529.17044	529.17096	13.5	0.986	119(100.00), 353(99.38), 233(63.30), 529(59.40)	√	√	√
	7.24	P	$C_{27}H_{31}O_{11}$	531.18609	531.18549	12.5	-1.126	179.03(100.00), 355.15(62.21), 299.09(19.94), 531.18(5.15)	√		
**M30**	7.36	N	$C_{27}H_{27}O_{12}$	543.1497	543.150270	14.5	-0.975	367(100.00), 543(12.03), 281(3.18)	√		
**M31**	7.49	P	$C_{27}H_{31}O_{11}$	531.18609	531.18579	12.5	-0.561	179(100.00), 531(34.67), 355(21.47), 299(12.67)	√		
	7.51	N	$C_{27}H_{29}O_{11}$	529.17044	529.17096	13.5	0.986	529(100.00), 119(52.89), 233(40.54), 353(23.06)			
**M32**	7.71	N	$C_{27}H_{31}O_{14}S$	611.14290	611.14319	12.5	0.470	119(100.00), 353(71.68), 611(60.17), 369(4.25), 531(1.51)			
**M33**	8.07	P	$C_{21}H_{21}O_{6}$	369.13326	369.13251	11.5	-2.045	369(100.00), 249(15.06), 179(4.16), 85(3.22)			
**M34**	8.24	N	$C_{26}H_{27}O_{11}$	515.15479	515.15570	13.5	-0.359	339(100.00), 515(34.79), 119(32.87)		√	√
	8.24	P	$C_{26}H_{29}O_{11}$	517.17044	517.17041	12.5	-2.175	341(100.00), 517(4.08), 179(3.34)	√	√	√
**M35**	8.30	N	$C_{21}H_{21}O_{8}S$	433.09626	433.09540	11.5	0.543	119(100.00), 233(67.75), 353(10.54), 433(76.72)			
	8.33	P	$C_{21}H_{23}O_{8}S$	435.11191	435.10977	10.5	-2.401	179(100.00), 435(45.03), 355(28.23), 299(22.31), 235(12.61), 121(1.29),			
**M36**	8.39	N	$C_{27}H_{29}O_{12}$	545.16535	545.16632	13.5	1.774	369(100.00), 119(75.73), 545(32.54), 250(5.78), 233(1.51)	√		√
	8.39	P	$C_{27}H_{31}O_{12}$	547.18100	547.18091	12.5	-0.279	371(32.58), 547(3.94), 179(3.21), 355(1.47), 235(0.96), 299(0.61)	√	√	
**M37**	8.42	P	$C_{21}H_{21}O_{6}$	369.13326	369.1322	11.5	-2.885	249(100.00), 369(51.07), 353(9.42), 231(6.52)			
**M38**	8.47	N	$C_{21}H_{21}O_{8}S$	433.09626	433.09601	11.5	1.952	119(100.00), 433(83.63), 233(71.85), 353(58.14)			
	8.47	P	$C_{21}H_{23}O_{8}S$	435.11191	435.1102	10.5	-1.413	179(100.00), 435(47.52), 355(26.88), 299(20.88), 235(11.17), 121(0.75)			
**M39**	8.65	N	$C_{21}H_{21}O_{9}S$	449.09058	449.09008	11.5	1.115	119(100.00), 369(83.93), 449(46.95), 80(34.52), 249(36.97)			
**M40**	8.83	N	$C_{21}H_{23}O_{8}S$	435.11081	435.11063	10.5	-0.425	353(100.00), 119(78.71), 233(43.17), 435(9.49),			
	8.86	P	$C_{21}H_{25}O_{8}S$	437.12646	437.12494	9.5	-3.488	179(100.00), 235(43.37), 299(17.34), 437(6.22), 355(1.60)			

**M41**	8.87	N	$C_{21}H_{23}O_{10}S$	467.10064	467.10071	10.5	0.1410	119(35.08), 467(6.44), 387(4.67), 247(2.51)			
**M42**	9.28	N	$C_{21}H_{23}O_8S$	435.11081	435.11194	10.5	2.586	353(100.00), 119(68.81), 233(35.24), 435(11.67), 96(2.77)	√		
	9.30	P	$C_{21}H_{25}O_8S$	437.12646	437.12604	9.5	-0.972	179(100.00), 235(44.98), 299(11.31), 437(9.22)			
**M43**	9.71	N	$C_{26}H_{27}O_{11}$	515.15479	515.15521	13.5	-1.310	339(100.00), 515(30.45), 119(27.85)		√	√
	9.71	P	$C_{26}H_{29}O_{11}$	517.17044	517.16968	12.5	-3.587	285(100.00), 165(67.89), 341(18.93), 517(12.74)		√	√
**M44**	9.99	N	$C_{27}H_{29}O_{14}S$	609.12725	609.12817	13.5	1.506	609(100.00), 353(86.40), 119(66.91), 233(63.16), 529(29.62),433(13.42)	√		
**M45**	10.03	N	$C_{21}H_{19}O_9S$	447.07443	447.0752	12.5	1.724	119(100.00), 447(23.81), 367(22.27), 96(1.24), 351(0.18)			
**M46**	10.37	P	$C_{21}H_{23}O_6$	371.14891	371.14767	10.5	-0.013	371(100.00), 179(24.36), 353(10.51), 547(7.77), 235(3.87)		√	
	10.36	N	$C_{21}H_{21}O_6$	369.13333	369.13436	11.5	1.504	369(100.00), 119(93.76), 250(3.65), 339(2.56)	√	√	√
**M47**	10.47	N	$C_{21}H_{21}O_7$	385.12818	385.12875	11.5	1.482	385(100.00), 119(64.76), 265(1.34), 149(1.08)			
**M48**	10.63	N	$C_{27}H_{29}O_{11}$	529.17044	529.17108	13.5	1.213	353(100.00), 119(87.24), 233(53.66), 529(35.90)	√	√	√
	10.63	P	$C_{27}H_{31}O_{11}$	531.18609	531.18542	12.5	-1.258	179(100.00), 299(32.08), 531(12.19), 355(8.72), 235(1.11)	√		
**M49**	10.79	P	$C_{21}H_{23}O_8$	403.13874	403.13831	10.5	-1.077	403(75.61), 355(5.95), 179(1.20), 299(1.06)			
**M50**	11.45	N	$C_{27}H_{29}O_{11}$	529.17044	529.17108	13.5	1.213	119(95.09), 353(67.23), 233(54.97), 529(41.65)	√		
	11.46	P	$C_{27}H_{31}O_{11}$	531.18609	531.18542	12.5	-1.258	179(100.00), 531(14.03), 299(4.75), 355(4.00), 235(1.33)	√		
**M51**	11.79	P	$C_{21}H_{21}O_6$	369.13326	369.13293	11.5	-0.907	249(100.00), 369(78.55), 353(31.27), 231(20.32), 351(4.32)			
**M52**	13.08	P	$C_{21}H_{23}O_8S$	435.11191	435.10992	10.5	-2.056	179(100.00), 435(12.91), 299(1.82), 355(1.14), 235(0.61)			
	13.09	N	$C_{21}H_{21}O_8S$	433.09626	433.09583	11.5	1.536	119(100.00), 353(65.57), 433(64.65), 233(63.70)	√		
**M53**	24.15	N	$C_{28}H_{31}O_{13}$	575.17592	575.17914	13.5	5.603	257(73.88), 433(24.22), 233(23.17), 119(15.49), 575(4.19)			
